# ^68^Ga-FAPI PET/CT: Biodistribution and Preliminary Dosimetry Estimate of 2 DOTA-Containing FAP-Targeting Agents in Patients with Various Cancers

**DOI:** 10.2967/jnumed.118.215913

**Published:** 2019-03

**Authors:** Frederik L. Giesel, Clemens Kratochwil, Thomas Lindner, Manfred M. Marschalek, Anastasia Loktev, Wencke Lehnert, Jürgen Debus, Dirk Jäger, Paul Flechsig, Annette Altmann, Walter Mier, Uwe Haberkorn

**Affiliations:** 1 Department of Nuclear Medicine, University Hospital Heidelberg, Heidelberg Germany; 2 ABX-CRO Advanced Pharmaceutical Services Forschungsgesellschaft m.b.H, Dresden, Germany; 3 Department of Radiation Oncology, University Hospital Heidelberg, Heidelberg, Germany; 4 Clinical Cooperation Unit Radiation Oncology, German Cancer Research Center, Heidelberg, Germany; 5 Department of Medical Oncology, National Center for Tumor Diseases, Heidelberg, Germany; 6 Clinical Cooperation Unit Nuclear Medicine, German Cancer Research Center, Heidelberg, Germany; and; 7 Translational Lung Research Center Heidelberg, German Center for Lung Research, Heidelberg, Germany

**Keywords:** radiotracer tissue kinetics, cancer-associated fibroblasts, PET/CT, biodistribution, dosimetry, fibroblast activation protein

## Abstract

Fibroblast activation protein (FAP) is overexpressed in cancer-associated fibroblasts of several tumor entities. The recent development of quinoline-based PET tracers that act as FAP inhibitors (FAPIs) demonstrated promising results preclinically and already in a few clinical cases. Consequently, these tracers are now applied in our hospital to amend the diagnostics of cancer patients facing the limitations of standard examinations. Here, we analyze the tissue biodistribution and preliminary dosimetry of 2 members of this new class of PET radiopharmaceutical. **Methods:** A preliminary dosimetry estimate for ^68^Ga-FAPI-2 and ^68^Ga-FAPI-4 was based on 2 patients examined at 0.2, 1, and 3 h after tracer injection using the QDOSE dosimetry software suit. Further PET/CT scans of tumor patients were acquired 1 h after injection of either ^68^Ga-FAPI-2 (*n* = 25) or ^68^Ga-FAPI-4 (*n* = 25); for 6 patients an intraindividual related ^18^F-FDG scan (also acquired 1 h after injection) was available. For the normal tissue of 16 organs, a 2-cm spheric volume of interest was placed in the parenchyma; for tumor lesions, a threshold-segmented volume of interest was used to quantify SUV_mean_ and SUV_max_. **Results:** Similar to literature values for ^18^F-FDG, ^68^Ga-DOTATATE, and ^68^Ga-PSMA-11, an examination with 200 MBq of ^68^Ga-FAPI-2 or ^68^Ga-FAPI-4 corresponds to an equivalent dose of approximately 3–4 mSv. After a fast clearance via the kidneys, the normal organs showed a low tracer uptake with only minimal changes between 10 min and 3 h after injection. In ^68^Ga-FAPI-2, the tumor uptake from 1 to 3 h after injection decreased by 75%, whereas the tumor retention was prolonged with ^68^Ga-FAPI-4 (25% washout). Regarding tumor-to-background ratios, at 1 h after injection both ^68^Ga-FAPI tracers performed equally. In comparison to ^18^F-FDG, the tumor uptake was almost equal (average SUV_max_, 7.41 for ^18^F-FDG and 7.37 for ^68^Ga-FAPI-2; not statistically significant); the background uptake in brain (11.01 vs. 0.32), liver (2.77 vs. 1.69), and oral/pharyngeal mucosa (4.88 vs. 2.57) was significantly lower with ^68^Ga-FAPI. Other organs did not relevantly differ between ^18^F-FDG and ^68^Ga-FAPI. **Conclusion:** FAPI PET/CT is a new diagnostic method in imaging cancer patients. In contrast to ^18^F-FDG, no diet or fasting in preparation for the examination is necessary, and image acquisition can potentially be started a few minutes after tracer application. Tumor-to-background contrast ratios were equal to or even better than those of ^18^F-FDG.

Fibroblast activation protein (FAP) is highly expressed in the stroma of several tumor entities. Especially breast, colon, and pancreatic carcinomas are characterized by a strong desmoplastic reaction, which means that 90% of the gross tumor mass can consist of stromal but not tumor cells.

Fibroblasts are present ubiquitously in the whole body and show dipeptidyl peptidase 4 expression but no or only a very low FAP expression. In contrast, cancer-associated fibroblasts are specifically characterized by the expression of FAP, which, unlike the closely related dipeptidyl peptidase 4, has not only exopeptidase activity but also endopeptidase activity; that is, proteins can be cleaved not only at their terminal end but at any postproline bond in the amino acid sequence (1). Thus, cancer-associated fibroblasts differ from normal fibroblasts by providing FAP as a target with a relatively high tumor-specific expression, and FAP inhibitors (FAPIs) have already been developed as cancer drugs (2,3).

Based on a quinoline-based FAP-specific inhibitor (2), a new class of radiopharmaceuticals was designed and found preclinically highly promising as molecular targeting imaging probes, and it is hoped that they also will be therapeutically useful (4,5). A few first-in-human cases demonstrated high-contrast tumor imaging and possible appropriateness as a pan-tumor agent (4,5).

Consequently, we are now increasingly using this tracer to amend the diagnostics of cancer patients who are facing the limitations of standard examinations.

Here, we approximated the radiation exposure of serial PET/CT using the ligands ^68^Ga-FAPI-2 and ^68^Ga-FAPI-4 and analyzed the normal-tissue biodistribution and tumor uptake of these ligands in comparison to the current standard, ^18^F-FDG.

## MATERIALS AND METHODS

### Patients

All patients gave written informed consent to undergo ^68^Ga-FAPI PET/CT following the regulations of the German Pharmaceuticals Act §13(2b). All patients were referred for the experimental diagnostics by their oncologists, who were facing an unmet diagnostic challenge that could not be solved sufficiently with standard diagnostic means. Examples of such challenges are insufficient tumor delineation for target-volume segmentation before external-beam radiotherapy, suspicion that lesions are false-negative on ^18^F-FDG, and the need to select target-positive patients for experimental last-line therapy with therapeutic FAPI conjugates. The data were analyzed retrospectively with the approval of the local ethics committee (approval S016/2018). Detailed patient characteristics are provided in Table 1.

**TABLE 1:** Patient Characteristics

Patient no.	Sex	Age	MBq	Diagnosis	Tracer
1	F	89	312	Breast cancer	^68^Ga-FAPI-2
2	M	55	298	Colorectal cancer	^68^Ga-FAPI-2, ^18^F-FDG
3	M	56	256	Cancer of unknown primary	^68^Ga-FAPI-2
4	M	64	336	Head and neck cancer	^68^Ga-FAPI-2
5	M	66	196	Head and neck cancer	^68^Ga-FAPI-2
6	F	64	202	Head and neck cancer	^68^Ga-FAPI-2
7	F	65	178	Head and neck cancer	^68^Ga-FAPI-2
8	M	59	325	Head and neck cancer	^68^Ga-FAPI-2
9	M	68	255	Head and neck cancer	^68^Ga-FAPI-2, ^18^F-FDG
10	M	70	212	Hepatocellular carcinoma	^68^Ga-FAPI-2
11	M	66	308	Liposarcoma	^68^Ga-FAPI-2
12	M	78	222	Non–small cell lung cancer	^68^Ga-FAPI-2, ^18^F-FDG
13	F	66	268	Non–small cell lung cancer	^68^Ga-FAPI-2
14	F	58	126	Esophagus cancer	^68^Ga-FAPI-2, ^18^F-FDG
15	M	70	134	Esophagus cancer	^68^Ga-FAPI-2
16	M	31	307	Pancreatic cancer	^68^Ga-FAPI-2, ^18^F-FDG
17	M	52	167	Pancreatic cancer	^68^Ga-FAPI-2
18	M	56	222	Pancreatic cancer	^68^Ga-FAPI-2
19	F	73	142	Pancreatic cancer	^68^Ga-FAPI-2
20	M	74	122	Prostate cancer	^68^Ga-FAPI-2
21	M	77	318	Prostate cancer	^68^Ga-FAPI-2
22	M	60	285	Renal cell carcinoma	^68^Ga-FAPI-2
23	M	77	225	Thyroid cancer	^68^Ga-FAPI-2, ^18^F-FDG
24	M	55	270	Thyroid cancer	^68^Ga-FAPI-2
25	F	60	238	Uterus cancer	^68^Ga-FAPI-2
26	F	57	263	Breast cancer	^68^Ga-FAPI-4
27	F	44	220	Colorectal cancer	^68^Ga-FAPI-4
28	M	66	286	Colorectal cancer	^68^Ga-FAPI-4
29	M	55	244	Colorectal cancer	^68^Ga-FAPI-4
30	M	46	247	Cancer of unknown primary	^68^Ga-FAPI-4
31	F	82	236	Head and neck cancer	^68^Ga-FAPI-4
32	M	51	263	Head and neck cancer	^68^Ga-FAPI-4
33	M	84	246	Hepatocellular carcinoma	^68^Ga-FAPI-4
34	M	77	299	Non–small cell lung cancer	^68^Ga-FAPI-4
35	F	58	217	Non–small cell lung cancer	^68^Ga-FAPI-4
36	M	64	255	Non–small cell lung cancer	^68^Ga-FAPI-4
37	F	56	250	Ovarian cancer	^68^Ga-FAPI-4
38	F	67	260	Pancreatic cancer	^68^Ga-FAPI-4
39	F	76	243	Pancreatic cancer	^68^Ga-FAPI-4
40	F	55	293	Pancreatic cancer	^68^Ga-FAPI-4
41	M	52	239	Pancreatic cancer	^68^Ga-FAPI-4
42	M	61	198	Pancreatic cancer	^68^Ga-FAPI-4
43	M	73	277	Pancreatic cancer	^68^Ga-FAPI-4
44	M	57	275	Pancreatic cancer	^68^Ga-FAPI-4
45	M	60	237	Pancreatic cancer	^68^Ga-FAPI-4
46	M	31	233	Pancreatic cancer	^68^Ga-FAPI-4
47	M	71	249	Prostate cancer	^68^Ga-FAPI-4
48	M	64	227	Prostate cancer	^68^Ga-FAPI-4
49	M	72	276	Thyroid cancer	^68^Ga-FAPI-4
50	F	27	204	Thyroid cancer	^68^Ga-FAPI-4

### Radiopharmaceuticals

**FIGURE 1.**
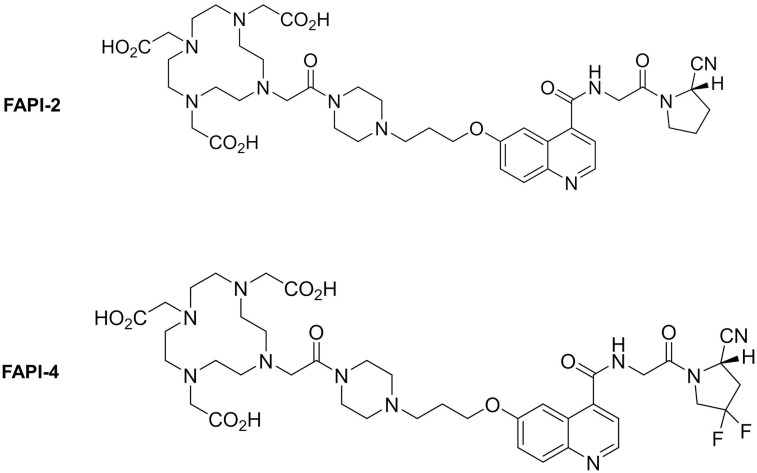
Molecular structure of ^68^Ga-FAPI-2 and ^68^Ga-FAPI-4.

Synthesis and labeling of ^68^Ga-FAPI-2 (4) and ^68^Ga-FAPI-4 (5) have already been described previously. ^18^F-FDG was obtained commercially (Life Radiopharma f-con GmbH). The chemical structures of ^68^Ga-FAPI-2 and ^68^Ga-FAPI-4 are provided in Figure 1.

### PET/CT Imaging

All imaging was performed on a Biograph mCT Flow scanner (Siemens). After unenhanced low-dose CT (130 keV, 30 mAs, CARE Dose [Siemens]; reconstructed with a soft-tissue kernel to a slice thickness of 5 mm), PET was acquired in 3-dimensional mode (matrix, 200 × 200) using FlowMotion (Siemens). The emission data were corrected for randoms, scatter, and decay. Reconstruction was performed with ordered-subset expectation maximization using 2 iterations and 21 subsets and Gauss filtration to a transaxial resolution of 5 mm in full width at half maximum. Attenuation correction was performed using the unenhanced low-dose CT data. The injected activity for the FAPI examinations was 122–336 MBq (details provided in Table 1), and the PET scans were started 1 h after injection.

To approximate the dosimetry, imaging was performed 10 min, 1 h, and 3 h after injection of 306 MBq of ^68^Ga-FAPI-2 in one patient and 258 MBq of ^68^Ga-FAPI-4 in another patient.

### Adverse Events

Standard vital parameters were checked by a medical technician between tracer application and up to 30 min after finishing the examination, and the patients were asked to report any abnormalities.

### Radiation Dosimetry Estimate

The dosimetry analysis was performed using the QDOSE dosimetry software suite (ABX-CRO). Kidneys, liver, spleen, urinary bladder content, red marrow, heart content, and remainder of body were included as source organs. Because of blood sampling failure due to poor vein conditions, the red marrow dose was approximated using the activity segmented with volume-of-interest technique in the PET scans. The non–tumor-affected lumbar vertebrae 5 (^68^Ga-FAPI-2) and 4 (^68^Ga-FAPI-4) were assumed to contain 2.46% of the total red marrow (6). Using QDOSE, all CT images were coregistered using an automatic deformable coregistration. PET images were coupled to the CT images of the corresponding imaging session. The PET images were transformed according to the transformation matrix of the coupled CT. The volumes of interest of all segmented source organs were drawn in the examination that had the best organ delineation and then were copied onto all other time points to calculate the time–activity curves. Monoexponential curve fitting was then applied to all organ time–activity curves. The cumulative activity Ã between time 0 and the first measured time point was calculated assuming a linear increase from 0 to the first measured activity. The Ã between the first measured time point and the last measured time point was integrated numerically using trapezoidal approximation. The Ã from the last measured time point to infinity was integrated using the fitted function. The total-body Ã was based on the injected activity and the calculated effective half-life in a total-field-of-view volume of interest, assuming that the limbs behave similar to the torso. This approach was chosen because some parts of the body were not in the field of view of the PET/CT device. The Ã values of total body and red marrow were added as organs into QDOSE. The Ã of the remainder of the body was then automatically calculated by subtracting all source-organ Ã values from the total-body Ã. All source-organ residence times were calculated by dividing the Ã by the injected activity. Absorbed and effective dose calculations were performed using the International Commission on Radiological Protection (ICRP)–endorsed IDAC-Dose 2.1 and IDAC-Dose 1.0 (7), which are integrated in QDOSE. In addition, the residence times of all included source organs and remainder of body were exported as an OLINDA case file for dose calculation in OLINDA 1.1 (8). Both IDAC-Dose 1.0 and OLINDA 1.1 are based on the Cristy–Eckerman stylized phantom series (9). IDAC-Dose 2.1 is based on the ICRP adult reference computational phantoms (10) and the ICRP-specific absorbed fractions (11). Organ masses were not adapted to individual subject organ masses.

### Biodistribution

The tracer biodistribution in patients was quantified by SUV_mean_ and SUV_max_ at 1 h after injection for ^68^Ga-FAPI-2, ^68^Ga-FAPI-4, and ^18^F-FDG. The interval between ^18^F-FDG and ^68^Ga-FAPI examinations was 9 d maximum, and no treatment change took place in between. For calculation of the SUV, circular regions of interest were drawn around the tumor lesions with focally increased uptake in transaxial slices and automatically adapted to a 3-dimensional volume of interest with e.soft software (Siemens) at a 40% isocontour. The normal organs were evaluated with a 1-cm-diameter (for the small organs [thyroid, parotid gland, myocardium, oral mucosa, and spinal cord]) to 2-cm-diameter (brain, muscle, liver, spleen, kidney, fat, aortic lumen content, and lung) sphere placed inside the organ parenchyma.

## RESULTS

### Adverse Events

All patients tolerated the examination well. No drug-related pharmacologic effects or physiologic responses occurred. All observed parameters (e.g., blood pressure, heart rate, and body temperature) remained normal and unchanged during injection and the 1.5 h of follow-up. No patient reported any symptoms.

### Dosimetry Estimate

**FIGURE 2.**
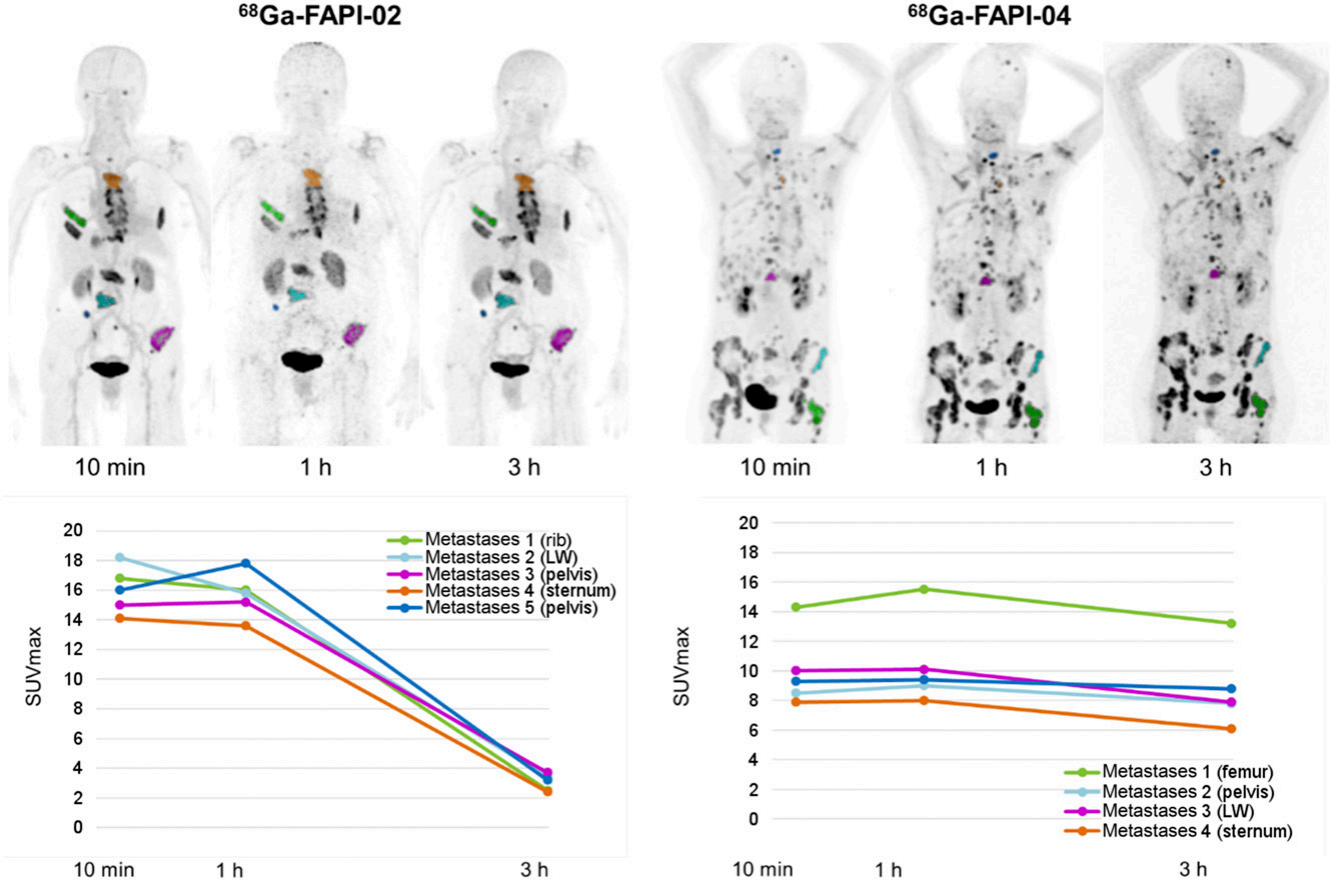
^68^Ga-FAPI-2 and ^68^Ga-FAPI-4 at different imaging time points (10 min, 1 h, and 3 h after injection) in 2 patients with metastasized breast cancer. Rapid tumor targeting and fast blood clearance are followed by long plateau phase without relevant change in image contrast (top). In comparison to ^68^Ga-FAPI-2, ^68^Ga-FAPI-4 is characterized by prolonged tumor retention time (bottom). LW = lumbar body.

Maximum-intensity projections of the PET scans used for source-organ segmentation are shown in Figure 2. The approximated dosimetry for the 2 patients is presented in Table 2. The effective dose of ^68^Ga-FAPI-2 was 1.80E−2 mSv/MBq calculated with OLINDA (1.82E−2 with IDAC1/ICRP60 and 1.79E−2 with IDAC2/ICRP103). The effective dose for ^68^Ga-FAPI-4 PET/CT was 1.64E−2 mSv/MBq calculated with OLINDA (1.66E−2 with IDAC1/ICRP60 and 1.35E−2 with IDAC2/ICRP103). If the delayed scan at 3 h after injection is omitted in clinical practice, the routine activity for a FAPI examination could be reduced to 200 MBq of ^68^Ga; consequently, the radiation dose of such a ^68^Ga-FAPI PET/CT scan would be 3–4 mSv.

**TABLE 2:** Dosimetry Estimate (OLINDA)

Site	^68^Ga-FAPI-2	^68^Ga-FAPI-4
Adrenals	1.23E−02	1.12E−02
Brain	9.54E−03	9.11E−03
Breasts	9.58E−03	8.88E−03
Gallbladder wall	1.19E−02	1.13E−02
Lower large intestine wall	1.23E−02	1.17E−02
Small intestine	1.19E−02	1.13E−02
Stomach wall	1.13E−02	1.06E−02
Upper large intestine wall	1.17E−02	1.11E−02
Heart wall	4.73E−02	2.02E−02
Kidneys	4.45E−02	4.43E−02
Liver	1.51E−02	1.46E−02
Lungs	1.09E−02	9.89E−03
Muscle	1.04E−02	9.91E−03
Ovaries	1.24E−02	1.19E−02
Pancreas	1.23E−02	1.13E−02
Red marrow	3.28E−02	2.08E−02
Osteogenic cells	2.94E−02	2.16E−02
Skin	9.01E−03	8.63E−03
Spleen	2.62E−02	1.05E−02
Testes	1.04E−02	1.01E−02
Thymus	1.15E−02	1.01E−02
Thyroid	1.03E−02	9.82E−03
Urinary bladder wall	8.89E−02	9.91E−02
Uterus	1.33E−02	1.30E−02
Total body	1.19E−02	1.09E−02
Effective dose (mSv/MBq)	1.80E−02	1.64E−02

### Biodistribution

The 2 patients examined 10 min to 3 h after injection demonstrated that both FAPI tracers rapidly reached their stable physiologic biodistribution. In normal tissue, changes between 10 min and 3 h after injection were minimal. Tumor uptake declined by a mean of 75% from 1 h to 3 h after injection using ^68^Ga-FAPI-2; less washout, only 25% (mean), between 1 h and 3 h after injection (i.e., longer tumor retention) was observed with ^68^Ga-FAPI-4 (Fig. 2, bottom). However, at 1 h after injection (the time point also chosen for comparison to ^18^F-FDG), both ^68^Ga-FAPI tracers performed equally with regard to tumor-to-background ratios.

**FIGURE 3.**
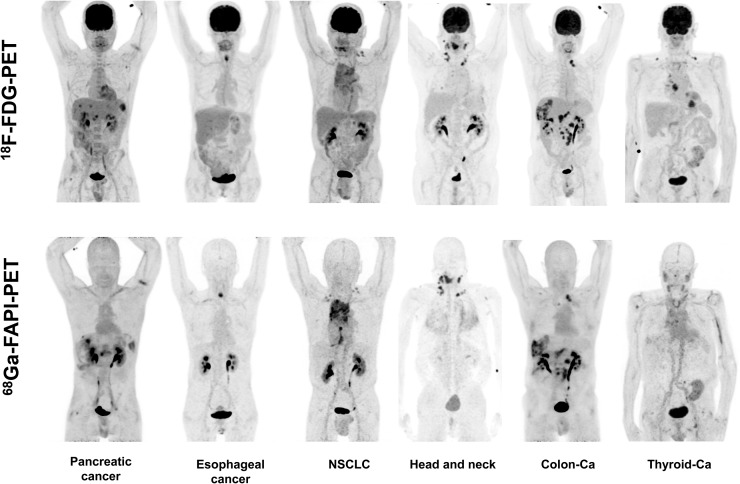
Intraindividual comparison of 6 patients with 6 different tumor entities undergoing ^18^F-FDG PET and ^68^Ga-FAPI PET imaging within less than 9 d. Five of 6 patients present similar strong tumor uptake with ^18^F-FDG and ^68^Ga-FAPI, and 3 of 6 could benefit from lower background in liver or pharyngeal mucosa. In contrast, iodine-negative thyroid cancer patient presented only minor ^68^Ga-FAPI tracer uptake compared with ^18^F-FDG. Ca = cancer; NSCLC = non–small cell lung cancer.

**FIGURE 4.**
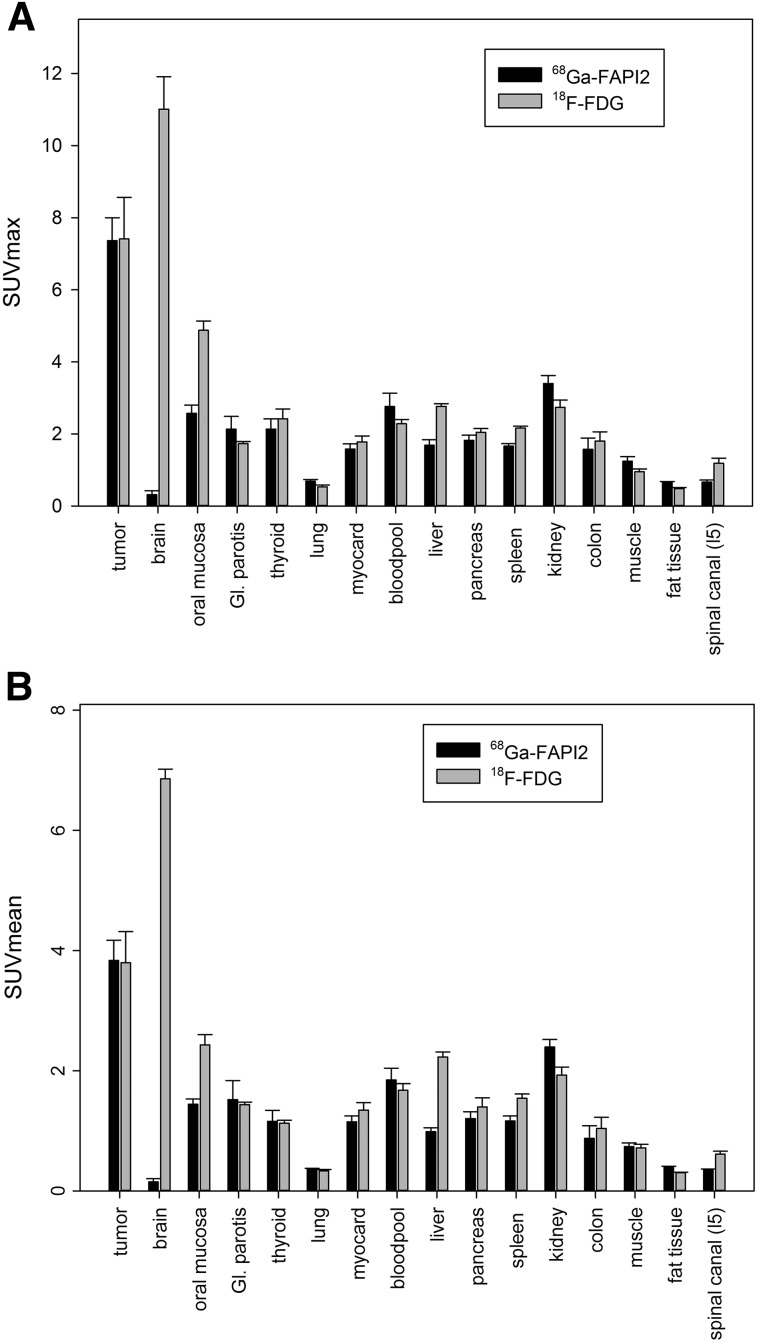
PET-based biodistribution analysis of 6 patients intraindividually comparing ^18^F-FDG PET and ^68^Ga-FAPI PET, imaged at 1 h after injection. GI = gastrointestinal.

The quantitative tumor uptake of FAPI PET was similar to that of the current oncologic PET standard of reference, ^18^F-FDG (average SUV_max_, 7.41 for ^18^F-FDG and 7.37 for ^68^Ga-FAPI-2; not statistically significant). In pancreatic, esophageal, lung, head and neck, and colorectal cancer, the quantitative tumor uptake was noninferior to that of ^18^F-FDG. In contrast, dedifferentiated thyroid cancer with flip-flop uptake of ^18^F-FDG was not accumulating ^68^Ga-FAPI (Fig. 3). Regarding background activity, the average SUV_max_ of ^68^Ga-FAPI-2 was significantly lower in brain (0.32 vs. 11.01), liver (1.69 vs. 2.77), and oral/pharyngeal mucosa (2.57 vs. 4.88), thus improving the contrast ratios for liver metastases of pancreatic and colorectal cancer and delineation of the esophageal cancer (Fig. 3). For all other organs, ^68^Ga-FAPI-2 presented no significant difference from ^18^F-FDG (Fig. 4A).

**FIGURE 5.**
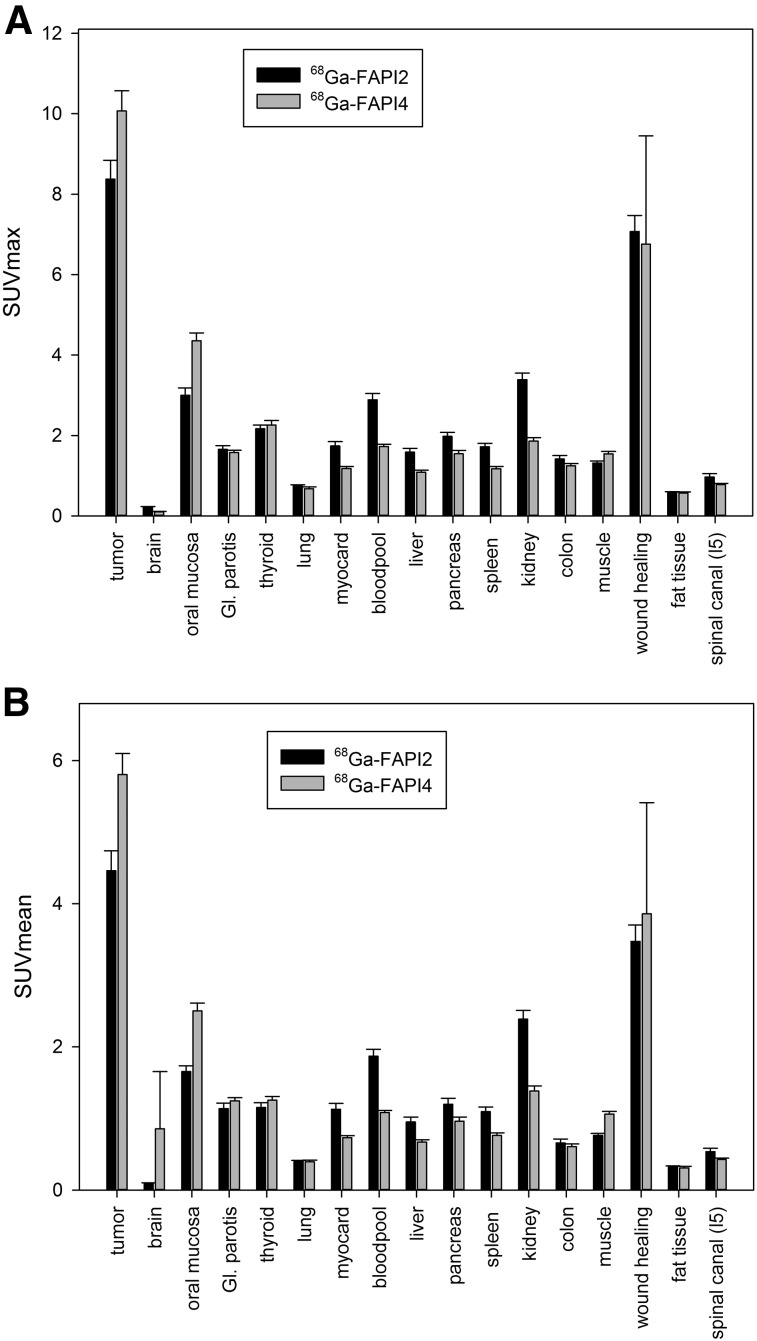
Interindividual comparison of 25 patients examined with ^68^Ga-FAPI-2 and 25 patients examined with ^68^Ga-FAPI-4 PET at 1 h after injection. GI = gastrointestinal.

Comparing uptake of ^68^Ga-FAPI-2 versus ^68^Ga-FAPI-4 by tumors, the average SUV_max_ presented no relevant differences (8.37 for ^68^Ga-FAPI-2 and 10.07 for ^68^Ga-FAPI-4; not statistically significant). Both tracers had unspecific uptake in locations of wound healing after surgical intervention (7.07 vs. 6.76; not statistically significant). Only minor differences between ^68^Ga-FAPI-2 and ^68^Ga-FAPI-4 were observed regarding normal-organ uptake (Fig. 4B).

## DISCUSSION

Recently, cancer-associated fibroblasts have been reported as a promising new multitumor target for small-molecule nuclear diagnostics (4,5). In this work, we initially approximated the radiation exposure of ^68^Ga-FAPI-2 and ^68^Ga-FAPI-4 PET and found it to be 1.4 and 1.8 mSv/100 MBq, respectively. We further analyzed the normal-tissue biodistribution of these ^68^Ga-FAPI ligands in comparison to ^18^F-FDG, the current standard in oncologic PET, and found comparable tumor uptake and, excepting a lower brain, liver, and oral mucosa uptake, comparable background uptake in normal organs.

The approximated dosimetry was limited by evaluating only one patient per tracer. However, the effective organ half-life and hence the radiation exposure is dominated more by the short physical half-life of ^68^Ga (68 min) than by the biologic half-life of the shuttle molecule, and thus it was no surprise that the effective dose of ^68^Ga-FAPI-2 and ^68^Ga-FAPI-4 PET (1.4–1.8 mSv/100 MBq) is similar to that of other ^68^Ga-based tracers, such as ^68^Ga-DOTATOC and ^68^Ga-DOTATATE (2.1 mSv/100 MBq) (12), ^68^Ga-PSMA-11 (1.6–2.4 mSv/100 MBq) (13,14), ^68^Ga-PSMA-617 (2.1 mSv/100 MBq), and ^18^F-FDG (2 mSv/100 MBq) (15), the current standards in oncologic PET.

It was not scope of this work to evaluate diagnostic accuracy such as the sensitivity and specificity of the new modality for a respective tumor entity. However, in a small cohort of challenging patients harboring various tumor diseases, the quantitative tumor uptake, as well as the background activity in most normal organs, was equal to that of ^18^F-FDG. A lower uptake in brain, liver, and oral–laryngeal mucosa might be promising for evaluation of brain or liver metastases, liver tumors, or head and neck tumors.

^68^Ga-FAPI PET/CT should also be considered complementary for tumor entities known to perform poorly with ^18^F-FDG, such as hepatocellular carcinoma or pancreatic cancer. Both have well-known limitations regarding ^18^F-FDG that are not yet completely covered by specific PET tracers (16,17).

We imaged several tumor entities with tumor-to-background ratios comparable to ^68^Ga-FAPI and ^18^F-FDG. We found high ^68^Ga-FAPI uptake in pancreatic cancer, esophageal cancer, non–small cell lung cancer, head and neck cancer, and colon cancer. In contrast, dedifferentiated thyroid cancer showed a low uptake or was ^68^Ga-FAPI–negative (Fig. 3). In this setting, the new imaging probes might benefit from their independence from blood sugar level, needing no dietary preparation. The rapid tumor uptake at 10 min after injection, as demonstrated in Figure 2, also indicates the possibility of early imaging. This could increase patient comfort because of a shorter waiting and scan time, which can be relevant in sick patients, and as a side aspect the radiation burden of the examination might be reduced if the injected activity can be reduced. Thus, the diagnostic performance of early versus late ^68^Ga-FAPI imaging should be evaluated more systematically in future studies. The possibility of early ^68^Ga-FAPI imaging would also avoid the 1-h uptake time with the patients resting, which is considered mandatory for ^18^F-FDG; consequently, ^68^Ga-FAPI PET could simplify the clinical workflow.

In contrast to ^18^F-FDG, the ^68^Ga-FAPI ligands contain DOTA as chelator, which can also be labeled with various therapeutic radionuclides. Taking into account the recent successes of radioligand therapy in neuroendocrine (18) and prostate (19) cancer, targeting FAP also presents a promising new approach in the treatment of these FAP-positive tumors. However, a further increase in tumor retention time, as in part already achieved in the development step from ^68^Ga-FAPI-2 to ^68^Ga-FAPI-4 (Fig. 2) (5), would still be required to refine the potential of FAPI-targeting radionuclide therapy, for example, with ^153^Sm or ^90^Y.

Similar to ^18^F-FDG, we observed some uptake in postsurgical wound healing because in this condition fibroblasts are also activated. Thus, we would not consider ^68^Ga-FAPI a more tumor-specific PET tracer than ^18^F-FDG. However, ^18^F-FDG is known to accumulate in acute inflammation, whereas FAP activation is typical of chronic inflammation already causing a fibrotic reaction (20,21). Localized FAP activation has also been reported in other diseases that are followed by tissue remodeling, such as myocardial infarction. Thus, ^68^Ga-FAPI PET/CT could play a complementary role to ^18^F-FDG in the field of chronic inflammatory cardiac diseases (22) or other diseases with tissue remodeling.

Although the discussed approaches were pursued, no final conclusion about their validity can be drawn from this first proof-of-concept investigation, which was intended to evaluate the dosimetry of ^68^Ga-labeled ^68^Ga-FAPI-2 and ^68^Ga-FAPI-4 diagnostics, identifying physiologic biodistribution and preliminary target validation in selected tumor entities. Further studies dedicated to evaluating the diagnostic performance in the respective clinically relevant settings are highly warranted.

## CONCLUSION

^68^Ga-FAPI PET/CT is a promising new diagnostic method for imaging various kinds of cancer, in particular pancreatic, head and neck, colon, lung, and breast cancer, with tumor-to-background contrast ratios equal to or even better than those of ^18^F-FDG. The favorable characteristics of the new ligands include fast tracer kinetics that seem appropriate for imaging patients even less than 1 h after injection; low background uptake in liver, oral mucosa, and brain; and independence from blood sugar. Because the ^68^Ga-FAPI tracers contain the universal DOTA-chelator, a theranostic approach—after labeling the ligand with an appropriate therapeutic radionuclide—also seems feasible.

## DISCLOSURE

Uwe Haberkorn, Anastasia Loktev, Thomas Lindner, and Walter Mier have filed a patent application for quinoline based FAP-targeting agents for imaging and therapy in nuclear medicine. No other potential conflict of interest relevant to this article was reported.

## References

[1] Hamson EJ Keane FM Tholen S Schilling O Gorrell MD Understanding fibroblast activation protein (FAP): substrates, activities, expression and targeting for cancer therapy. Proteomics Clin Appl. 2014;8:454–463. 24470260 10.1002/prca.201300095

[2] Jansen K Heirbaut L Cheng JD selective inhibitors of fibroblast activation protein (FAP) with a (4-quinolinoyl)-glycyl-2-cyanopyrrolidine scaffold. ACS Med Chem Lett. 2013;4:491–496. 24900696 10.1021/ml300410d PMC4027141

[3] Poplawski SE Lai JH Li Y Identification of selective and potent inhibitors of fibroblast activation protein and prolyl oligopeptidase. J Med Chem. 2013;56:3467–3477. 23594271 10.1021/jm400351a PMC4059180

[4] Loktev A Lindner T Mier W A tumor-imaging method targeting cancer-associated fibroblasts. J Nucl Med. 2018;59:1423–1429. 29626120 10.2967/jnumed.118.210435 PMC6126438

[5] Lindner T Loktev A Altmann A Development of quinoline-based theranostic ligands for the targeting of fibroblast activation protein. J Nucl Med. 2018;59:1415–1422. 29626119 10.2967/jnumed.118.210443

[6] Hindorf C Glatting G Chiesa C Linden O Flux G EANM Dosimetry Committee guidelines for bone marrow and whole-body dosimetry. Eur J Nucl Med Mol Imaging. 2010;37:1238–1250. 20411259 10.1007/s00259-010-1422-4

[7] Andersson M Johansson L Eckerman K Mattsson S IDAC-Dose 2.1, an internal dosimetry program for diagnostic nuclear medicine based on the ICRP adult reference voxel phantoms. EJNMMI Res. 2017;7:88. 29098485 10.1186/s13550-017-0339-3 PMC5668221

[8] Stabin MG Sparks RB Crowe E OLINDA/EXM: the second-generation personal computer software for internal dose assessment in nuclear medicine. J Nucl Med. 2005;46:1023–1027. 15937315

[9] Cristy M Eckerman KF *Specific Absorbed Fractions of Energy at Various Ages from Internal Photon Sources.* Oak Ridge, TN: Oak Ridge National Laboratory; 1987. ORNL/TM-8381/V1–V7.

[10] Menzel HG Clement C DeLuca P ICRP publication 110: realistic reference phantoms—an ICRP/ICRU joint effort. A report of adult reference computational phantoms. Ann ICRP. 2009;39:1–164. 10.1016/j.icrp.2009.09.001 19897132

[11] Bolch WE Jokisch D Zankl M ICRP publication 133: the ICRP computational framework for internal dose assessment for reference adults: —specific absorbed fractions. Ann ICRP. 2016;45:5–73. 10.1177/0146645316661077 29749258

[12] Sandström M Velikyan I Garske-Román U Comparative biodistribution and radiation dosimetry of ^68^Ga-DOTATOC and ^68^Ga-DOTATATE in patients with neuroendocrine tumors. J Nucl Med. 2013;54:1755–1759. 23929824 10.2967/jnumed.113.120600

[13] Pfob CH Ziegler S Graner FP Biodistribution and radiation dosimetry of ^68^Ga-PSMA HBED CC: a PSMA specific probe for PET imaging of prostate cancer. Eur J Nucl Med Mol Imaging. 2016;43:1962–1970. 27207281 10.1007/s00259-016-3424-3

[14] Afshar-Oromieh A Hetzheim H Kübler W Radiation dosimetry of ^68^Ga-PSMA-11 (HBED-CC) and preliminary evaluation of optimal imaging timing. Eur J Nucl Med Mol Imaging. 2016;43:1611–1620. 27260521 10.1007/s00259-016-3419-0

[15] Johansson L Mattsson S Nosslin B Leide-Svegborn S Effective dose from radiopharmaceuticals. Eur J Nucl Med. 1992;19:933–938. 1308762 10.1007/BF00175858

[16] Yun M Bang SH Kim JW Park JY Kim KS Lee JD The importance of acetyl coenzyme A synthetase for ^11^C-acetate uptake and cell survival in hepatocellular carcinoma. J Nucl Med. 2009;50:1222–1228. 19617323 10.2967/jnumed.109.062703

[17] Strobel O Büchler MW Pancreatic cancer: FDG-PET is not useful in early pancreatic cancer diagnosis. Nat Rev Gastroenterol Hepatol. 2013;10:203–205. 23478386 10.1038/nrgastro.2013.42

[18] Strosberg J El-Haddad G Wolin E Phase 3 trial of ^177^Lu-Dotatate for midgut neuroendocrine tumors. N Engl J Med. 2017;376:125–135. 28076709 10.1056/NEJMoa1607427 PMC5895095

[19] Hofman MS Violet J Hicks RJ ^177^Lu-PSMA-617 radionuclide treatment in patients with metastatic castration-resistant prostate cancer (LuPSMA trial): a single-centre, single-arm, phase 2 study. Lancet Oncol. 2018;19:825–33. 29752180 10.1016/S1470-2045(18)30198-0

[20] Egger C Cannet C Gérard C Effects of the fibroblast activation protein inhibitor, PT100, in a murine model of pulmonary fibrosis. Eur J Pharmacol. 2017;809:64–72. 28506908 10.1016/j.ejphar.2017.05.022

[21] Uitte de Willige S Malfliet JJ Janssen HL Leebeek FW Rijken DC Increased N-terminal cleavage of alpha-2-antiplasmin in patients with liver cirrhosis. J Thromb Haemost. 2013;11:2029–2036. 24034420 10.1111/jth.12396

[22] Tillmanns J Hoffmann D Habbaba Y Fibroblast activation protein alpha expression identifies activated fibroblasts after myocardial infarction. J Mol Cell Cardiol. 2015;87:194–203. 26319660 10.1016/j.yjmcc.2015.08.016

